# Newly Shaped Intra‐Aortic Balloons Improve the Performance of Counterpulsation at the Semirecumbent Position: An In Vitro Study

**DOI:** 10.1111/aor.12791

**Published:** 2016-08-16

**Authors:** Christina Kolyva, John R. Pepper, Ashraf W. Khir

**Affiliations:** ^1^ Department of Mechanical Engineering, Brunel UniversityUxbridge; ^2^ National Heart and Lung InstituteImperial College LondonLondonUK

**Keywords:** Intra‐aortic balloon pump, Counterpulsation, Balloon shape, Balloon efficacy, Semirecumbent, Hemodynamics

## Abstract

The major hemodynamic benefits of intra‐aortic balloon pump (IABP) counterpulsation are augmentation in diastolic aortic pressure (*P*_aug_) during inflation, and decrease in end‐diastolic aortic pressure (ΔedP) during deflation. When the patient is nursed in the semirecumbent position these benefits are diminished. Attempts to change the shape of the IAB in order to limit or prevent this deterioration have been scarce. The aim of the present study was to investigate the hemodynamic performance of six new IAB shapes, and compare it to that of a traditional cylindrical IAB. A mock circulation system, featuring an artificial left ventricle and an aortic model with 11 branches and physiological resistance and compliance, was used to test one cylindrical and six newly shaped IABs at angles 0, 10, 20, 30, and 40°. Pressure was measured continuously at the aortic root during 1:1 and 1:4 IABP support. *Shape 2* was found to consistently achieve, in terms of absolute magnitude, larger ΔedP at angles than the cylindrical IAB. Although ΔedP was gradually diminished with angle, it did so to a lesser degree than the cylindrical IAB; this diminishment was only 53% (with frequency 1:1) and 40% (with frequency 1:4) of that of the cylindrical IAB, when angle increased from 0 to 40°. During inflation *Shape 1* displayed a more stable behavior with increasing angle compared to the cylindrical IAB; with an increase in angle from 0 to 40°, diastolic aortic pressure augmentation dropped only by 45% (with frequency 1:1) and by 33% (with frequency 1:4) of the drop reached with the cylindrical IAB. After compensating for differences in nominal IAB volume, *Shape 1* generally achieved higher *P*_aug_ over most angles. Newly shaped IABs could allow for IABP therapy to become more efficient for patients nursed at the semirecumbent position. The findings promote the idea of personalized rather than generalized patient therapy for the achievement of higher IABP therapeutic efficiency, with a choice of IAB shape that prioritizes the recovery of those hemodynamic indices that are more in need of support in the unassisted circulation.

Over the past five decades the intra‐aortic balloon pump (IABP) has established a successful track record in delivering minimally invasive mechanical circulatory support in various clinical scenarios 1, 2, 3. The IAB is inflated at aortic valve closure and deflated prior to aortic valve opening, with the electrocardiogram (ECG) or the aortic pressure waveform used for the synchronization of the native heart and the device.

The main direct hemodynamic benefits of the IABP are augmentation in diastolic aortic pressure (*P*_aug_ao_) due to inflation and reduction in end‐diastolic aortic pressure (ΔedP_ao_) due to deflation. These effects are achieved through blood volume displacement upstream the IAB tip, with blood pushed toward the aortic arch during inflation and sucked away from the aortic arch during deflation. It is broadly accepted that *P*_aug_ao_ and ΔedP_ao_ stimulate enhanced coronary blood flow and improved unloading of the left ventricle (LV), respectively, causing in turn an increase in myocardial oxygen supply and decrease in demand.

Nursing critically ill patients in the semirecumbent position (head and torso inclined at angles of up to 45°) is widely adopted as a potential prophylactic measure against ventilator‐associated pneumonia 4. In this clinical scenario, the effects of the upright positioning on other physiological mechanisms are often not fully appreciated. Angulation has a pronounced effect on the performance of the IABP and is associated with changes in the inflation and deflation pattern of the IAB 5, 6, 7; inflation is generally reported to become faster and deflation to become slower at an angle 6. Moreover, while at the horizontal position the IAB inflates and deflates uniformly along its length, at angled positions inflation starts from the tip and progresses toward the base and, following exactly the opposite pattern, deflation progresses from base to tip. These differences in the pattern of inflation and deflation at angled positions are accompanied by adverse hemodynamic changes, such as drop in diastolic aortic pressure augmentation 6, 8, diastolic coronary flow 5, and, if the absolute magnitude is taken into account, drop in end‐diastolic aortic pressure reduction as well 6, 7, 8.

Relatively few efforts to implement design changes on the IAB in order to increase blood volume displacement during inflation and deflation, and thus improve the overall counterpulsation benefit, have been made, despite the IABP being clinically used for several decades. The addition of an umbrella‐type of valve at the base of the IAB catheter was proposed as a solution for preventing the retrograde abdominal aortic flow that is generated during deflation, from interfering with the concomitant antegrade ascending aortic flow which is responsible for end‐diastolic LV unloading 9. A similar rationale lies behind the design of a dual‐chamber IAB; a distal spherical chamber, connected in‐series to the base of a cylindrical IAB chamber, is inflated early in diastole, occluding the aorta and thus forcing all blood volume displaced during the simultaneous inflation of the cylindrical IAB to move in its entirety toward the aortic root 10. Various other multichamber IAB designs have emerged over the years, aiming to improve inflation and deflation IABP efficacy through the operating flexibility allowed by chambers connected in‐series; some designs consisted of chambers constructed to fit different parts of the aorta 11, while others allowed inflation/deflation of each chamber to be controlled independently and thus enabled full control over the sequence in which the chambers inflated/deflated (from base to tip or from tip to base) 12. An enhanced IAB consisting of internal and external IABs that are both connected to the same IABP driving system has also been tested; the internal IAB is positioned in the descending aorta and the external IAB is positioned inside a chamber, the output of which drains through a conduit just upstream the internal IAB tip 13. This design provided added capacity for early diastolic aortic pressure augmentation and end‐diastolic LV unloading, delivered directly in the ascending aorta. A shorter and wider version of the cylindrical IAB that is currently used clinically has also been tested very recently with favorable hemodynamic results 14.

A complete change in IAB shape for the enhancement of intra‐aortic counterpulsation performance was proposed in 2000 by Meyns et al. 15, who designed an IAB catheter intended to be advanced to the ascending rather than the descending aorta, via the femoral artery. The shape of this IAB was such that it enabled distal occlusion of the ascending aorta during inflation, in order to support augmentation in aortic root pressure

In an attempt to tackle the setback of the deterioration in IABP performance observed at angled positions, two new conical IAB shapes were presented in 2013 by our group, one with increasing (TID) and one with decreasing (TDD) diameter from base to tip 16. These shapes were designed in order to essentially counteract the negative effects of the changes in the inflation and deflation pattern of the cylindrical IAB that occur with angle. Following in vitro testing these new shapes, and in particular the TDD, showed potential for more stable and improved hemodynamic performance compared to the cylindrical IAB at angles. However, these new IABs required redesigning in order to fit in the physiological space that is available in the human descending aorta.

The aim of the present study is to investigate the hemodynamic performance of six new IABs, and compare it to that of a cylindrical IAB. We also aimed to identify the shapes that had the best hemodynamic performance during inflation and during deflation.

## Materials and Methods

### Balloons

**Figure 1:**
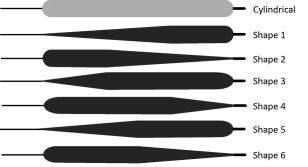
Schematic diagram of the cylindrical IAB and the six newly shaped IABs that were tested. Configurations with increasing diameter from base to tip and 2/3, 1/3, and 1/2 conical segments correspond to *Shape 1*, *Shape 3*, and *Shape 5*, respectively. Configurations with decreasing diameter from base to tip and 2/3, 1/3, and 1/2 conical segments correspond to *Shape 2*, *Shape 4*, and *Shape 6*, respectively. The diagram serves only as a schematic representation of the different shapes and the relative dimensions between the IABs are not precise.

Six newly shaped IABs were manufactured by Teleflex (Reading, PA, USA) according to the standard production process followed for all commercial IAB products of the company, and using the same materials. The new shapes were based on the traditional cylindrical shape, but 1/3, 1/2, or 2/3 of their total length was substituted by a conical segment, with the joining edge between the cylindrical and conical segment blunted in order to minimize the possibility of additional hemolysis risk, as shown in Fig. 1. Each of the above three newly shaped IAB membranes produced a lanceolate (“*lance*”) and an oblanceolate (“*oblance*”) configuration (this terminology is borrowed from botanology, where it is used for the description of leaf shapes), by mounting the tip and catheter in opposite ways. Overall, in the resulting new IABs the diameter of the cylindrical segment varied from 2.4 to 2.7 cm and the total length of the membrane varied from 23.0 to 23.6 cm. Oblance configurations with 2/3, 1/3, and 1/2 conical segments corresponded to *Shape 1*, *Shape 3*, and *Shape 5*, respectively (Fig. 1). Lance configurations with 2/3, 1/3, and 1/2 conical segments corresponded to *Shape 2*, *Shape 4*, and *Shape 6*, respectively (Fig. 1). The nominal volume of these balloons, when operating at transmural pressure of 110 mm Hg, was 34 cc.

A standard 40 cc cylindrical IAB (Fiberoptix 8Fr, Teleflex) was used as a reference for hemodynamic performance comparisons.

### Experimental setup

**Figure 2:**
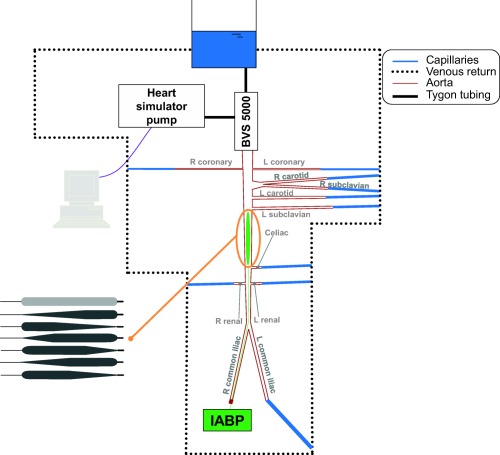
Schematic representation of the mock circulation system. The “aortic root” of the aortic model was connected to the “left ventricle” of an extracorporeal left ventricular assist device (BVS5000). Physiological distribution of terminal resistance was implemented across the model with capillary tubes of different size fitted at the outlets of all “arterial” branches. The ends of the branches were attached to a common tube that simulated the venous system and was connected to an overhead reservoir open to the air. The loop was closed by connecting the “left atrium” to the reservoir, which in turn was filled with enough water to provide atrial pressure of 15 mm Hg. The artificial heart was driven by a heart simulator pump. The system was assembled on a tilting platform and could be operated at angles 0–70° to the horizontal.

The IABs were tested in a mock circulation system (MCS) which was assembled according to the methods described in Kolyva et al. 17 and is shown in Fig. 2. Briefly, the “aortic root” of a real size silicone rubber aortic model (HemoLab, Eindhoven, The Netherlands) with 11 main branches (left and right coronary, left and right carotid, left and right subclavian, celiac, left and right renal, and left and right common iliac arteries) was connected to the “LV” of an extracorporeal left ventricular assist device (BVS 5000 blood pump, Abiomed Inc., Danvers, MA, USA). The device simulated the LV and the left atrium, and incorporated aortic and mitral valve equivalents as well. The total arterial compliance of the aortic model was measured in conformance with the method reported in Ref. 17 and was 1.67 mL/mm Hg, which was considered ample for the representation of the human aorta 18. Physiological distribution of terminal resistance according to published data 18 was implemented across the model with capillary tubes of different size fitted at the outlets of all branches. The ends of the “aortic” branches were attached to a common tube that simulated the venous system and was connected to an overhead reservoir open to the air. The loop was closed by connecting the “left atrium” to the reservoir, which in turn was filled with enough water to provide atrial pressure of 15 mm Hg. The use of water as flowing medium in setups used for IABP testing is common in the literature 17, 19, 20.

In the clinical setting the BVS 5000 is driven by an external console through a pneumatic (air) line. However, this console did not allow full operator control over the generated flow profile, neither in terms of time nor in terms of volume parameters. Therefore, we used a positive displacement pump (Placepower, Gorleston, UK) instead, to drive with water the artificial heart of the MCS. This combination essentially enabled us to simulate reproducibly any physiological flow profile, irrespective of IAB performance or of the operating angle of the MCS. The pump was set to provide stroke volume of 50 mL, at a heart rate of 58 bpm with diastolic time fraction (DTF) of 55% (cardiac output 2.9 L/min).

The MCS was assembled on a tilting platform and could be operated at a range of angles (0–70°), to simulate the semirecumbent position.

### IABP settings

The IABs were advanced in the descending aorta via the “right common iliac artery” and positioned with the tip just distal to the “left subclavian” branch, according to conventional clinical practice (Fig. 2). The IABs were connected to an IABP (AutoCAT 2 WAVE, Teleflex) via the helium line that matched more closely their nominal volume.

The IABP was set to counterpulsate with the artificial heart of the MCS, using the “ECG” output of the hydraulic pump for synchronizing the two devices. The IABP was set at operating mode “OPERATOR” and, based on the shape of the “ECG,” trigger mode “VPACE” was selected. The onset of inflation was set at 40% and the onset of deflation at 75% of the duration of the heart cycle. This enabled the onset of inflation to coincide with the closure of the “aortic valve,” and the onset of deflation to occur early enough to allocate adequate time for a more lengthy deflation at an angle 6, while a considerable reduction in end‐diastolic “aortic root” pressure could still be effected. The end of systole was programmed at 45% in the hydraulic pump. However, due to the delay involved in the translation of the electronic triggering of IAB inflation into shuffling helium in the IAB 21, inflation was required to be triggered at 40% in order for the IAB to start inflating at “aortic valve” closure. All IABs were inflated at full augmentation volume.

### Measurements and protocol

**Figure 3:**
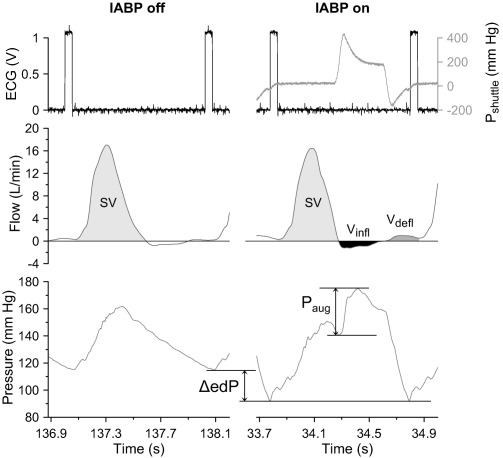
Measurements obtained with the cylindrical IAB positioned at 0°, when the IABP was off (left panel) and during 1:1 IABP support (right panel). Top panels: “ECG” and shuttle gas pressure (*P*_shuttle_) waveforms; middle panels: “aortic” flow waveform; and bottom panels: “aortic” pressure waveform. The flow integrations that yielded stroke volume (SV), volume displaced upstream the IAB tip due to inflation (*V*_infl_) and volume displaced upstream the IAB tip due to deflation (*V*_defl_) are shown in the middle panels. The way reduction in end‐diastolic “aortic” pressure (ΔedP) and diastolic “aortic” pressure augmentation (*P*_aug_) were derived is illustrated in the bottom panels.

Simultaneous pressure (*P*) and flow (*Q*) measurements were obtained at the “aortic root” of the MCS, just distal to the “coronary arteries.” *P* was measured with the tip sensor of a solid‐state, 3Fr dual‐pressure‐sensor‐equipped catheter (Mikro‐Tip, Millar Inc., Houston, TX, USA). *Q* was measured using a 32‐mm ultrasonic perivascular flowprobe (Transonic Systems Inc., Ithaca, NY, USA). Shuttle gas pressure (*P*_shuttle_) from the output of the IABP (as a reference for IAB inflation/deflation timing) and the “ECG” from the output of the hydraulic pump were also obtained. All data were sampled at 1000 Hz using a LabVIEW‐based acquisition system (National Instruments Corporation Ltd., Newbury, UK). Typical example waveforms recorded with the cylindrical IAB positioned at 0° are provided in Fig. 3.

Recordings were made with the MCS at angles 0, 10, 20, 30, and 40° to the horizontal. For each angle, each IAB was tested sequentially at assistance frequencies 1:1 and 1:4, as representative of full and weaning IABP support frequencies, respectively. For every combination of assistance frequency, IAB shape, and angle, each recording consisted of approximately 1 min with the IABP on for 1:1 and 2 min for 1:4 assistance frequencies, followed by 1 min with the IABP at standby (“baseline”), and was repeated twice. All beats recorded with the IABP on at frequency 1:1, but only every fourth beat recorded with the IABP on at frequency 1:4, will show the effect of counterpulsation/IABP support (“*assisted*” beats). The latter frequency is not merely a “time‐diluted” mode of 1:1; there is evidence suggesting that the therapeutic effect provided by a 1:4 assisted beat does not match the hemodynamic benefits delivered by a 1:1 assisted beat 22.

### Data analysis

All data were processed in Matlab (version R2011b, MathWorks, Natick, MA, USA). For every recording, the “ECG R‐peaks” were detected and used for marking the onset of each “cardiac” cycle. Subsequently, segments of 10 consecutive baseline beats and 10 consecutive beats with the IABP on (40 for support frequency 1:4) were selected and stored separately for further analysis. The *P* and *Q* signals were smoothed offline with a cubic 31‐point Savitzky‐Golay filter.

Diastolic *P* augmentation (*P*_aug_) and end‐diastolic *P* reduction (ΔedP) were calculated for the assisted beats and taken as indicators of the benefit of IAB inflation and deflation, respectively. *P*_aug_ was defined as the difference between peak diastolic *P* and *P* at the dicrotic notch/onset of inflation (Fig. 3). Subtraction from the end‐diastolic *P* (edP) of each assisted beat, of the mean edP of the corresponding 10 baseline beats, yielded ΔedP (Fig. 3).

For each assisted beat, stroke volume (SV), volume pushed upstream the IAB tip due to inflation (*V*_infl_) and volume pulled from upstream the IAB tip due to deflation (*V*_defl_) were calculated by integration of the *Q* signal with respect to time (Fig. 3). For the determination of SV, positive systolic *Q* was integrated, while the derivation of *V*_infl_ required the integration of the subsequent negative diastolic *Q*. Integration of the positive diastolic *Q* yielded *V*_defl_. For each baseline beat, SV was calculated in the same way as for the assisted beat (Fig. 3). Parameters *V*_infl_ and *V*_defl_ were used as secondary markers of IAB inflation and deflation benefit, while SV enabled the monitoring of the beat‐to‐beat consistency of the hydraulic pump.

For the above hemodynamic parameters, averages were derived from the 20 assisted beats that were analyzed in total for each combination of assistance frequency, IAB shape, and angle. To account for the difference in nominal IAB volume between the cylindrical and the newly shaped IABs, the averages of the main IABP parameters of interest were normalized with respect to the nominal IAB volume and yielded parameters norm_*P*_aug_, norm_ΔedP, norm_*V*_infl_, and norm_*V*_defl_.

### Statistics

The values reported are mean ± standard deviation. The statistical analysis was performed in MLwiN (version 2.33, Centre for Multilevel Modelling, University of Bristol, Bristol, UK). For *P*_aug_, ΔedP, *V*_infl_, and *V*_defl_, the following comparisons were made on the assisted beats: (i) separately for each IAB, values at 0° were compared to values at each of the other angles and (ii) separately for each angle, values with the cylindrical IAB were compared to values with each of the other IABs. These comparisons were made separately for the two assistance frequencies; it was outside the scope of this paper to compare the assistance frequencies to each other.

The statistical comparisons were performed with mixed model analysis. A statistical model was generated, in which the individual processed beats and the recording repetitions were considered random effects (for each combination of assistance frequency, IAB shape, and angle). In contrast, the different angles and IAB shapes were considered fixed effects and all interaction terms between them were taken into account, if they were significant (*P* < 0.2). Two such models were built, one for each assistance frequency. Each model was based on all 700 assisted beats that were analyzed in total for its corresponding assistance frequency. Allowances for a possible effect of mean *P* were also made, to compensate for the possibility that mean *P* may vary somewhat (within the range of ± 10 mm Hg) when the MCS operated at different angles. Statistical significance was assumed at *P* < 0.05.

## Results

### End‐diastolic pressure reduction

**Figure 4:**
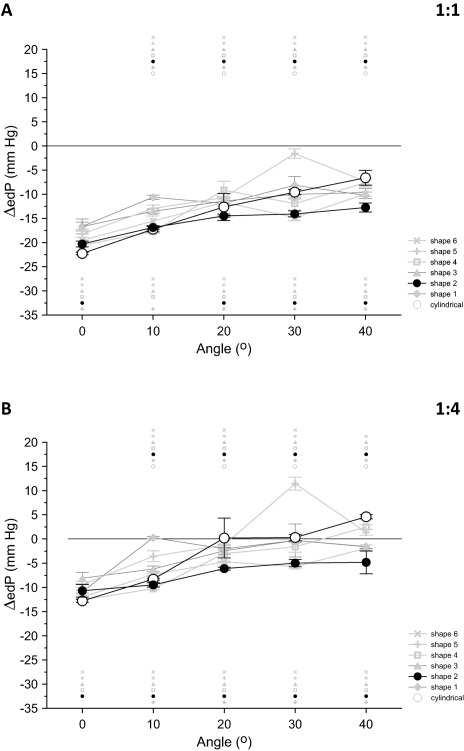
Average data (A) at frequency 1:1 and (B) at frequency 1:4, of the changes in end‐diastolic “aortic” pressure reduction (ΔedP) that took place when angle increased from 0 to 40° in increments of 10°. Data are shown separately for each IAB, each point is the average of 20 beats, and the error bars indicate standard deviations. For each IAB, the small matching symbols on top of each plot indicate statistically significant differences between 0° and each of the other angles. Similarly, the small matching symbols under each plot denote statistically significant differences compared to the cylindrical IAB. Statistical significance was assumed at *P* < 0.05.

Figure 4A,B presents, for each of the seven IABs that were tested, the changes observed in ΔedP with variations in angle for support frequency 1:1 and 1:4, respectively.

For both assistance frequencies the reduction in edP that could be achieved with the cylindrical IAB, compared to baseline, was gradually diminished with increasing angle. For frequency 1:1, ΔedP was 70% less in absolute magnitude at 40° compared to 0° (−6.6 ± 1.5 mm Hg at 40° vs. −22.3 ± 0.3 mm Hg at 0°, *P* < 0.05) and for frequency 1:4 this percentage was 136% (4.6 ± 0.5 mm Hg at 40° vs. −12.8 ± 0.3 mm Hg at 0°, *P* < 0.05). At 1:4, ΔedP was not only diminished at 40°, but was in fact positive, indicating that edP increased during IABP support compared to baseline.

Among the newly shaped IABs tested, *Shape 2* consistently achieved, in terms of absolute magnitude, larger ΔedP at angles than the cylindrical IAB. This ΔedP was gradually diminished by angle, but to a lesser degree compared to the cylindrical IAB. *Shape 2* produced a 37% deterioration in ΔedP with frequency 1:1 (−12.7 ± 0.9 mm Hg at 40° vs. −20.3 ± 0.6 mm Hg at 0°, *P* < 0.05) and 55% deterioration with frequency 1:4 (−4.8 ± 2.4 mm Hg at 40° vs. −10.7 ± 1.3 mm Hg at 0°, *P* < 0.05) at 40° compared to 0°. Both for 1:1 and 1:4 ΔedP with *Shape 2* was statistically significantly different from ΔedP with the cylindrical IAB at all angles but one (10° at 1:1).

**Figure 5:**
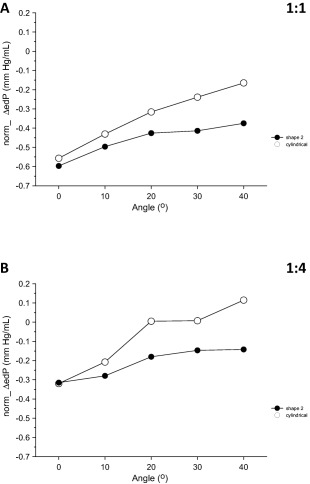
Changes in normalised end‐diastolic “aortic” pressure reduction (norm_ΔedP) when angle increased from 0 to 40° in increments of 10° (A) at frequency 1:1 and (B) at frequency 1:4. Data are shown for the cylindrical IAB and for *Shape 2*. Each point was derived by dividing the corresponding average ΔedP with the nominal IAB volume.

It is noted that the superiority of *Shape 2* described above is already evident before any normalization for nominal IAB volume is done. Figure 5A,B demonstrates that after the advantage in favor of the cylindrical IAB was removed, the enhanced performance of *Shape 2* became even more pronounced.

### Diastolic pressure augmentation

**Figure 6:**
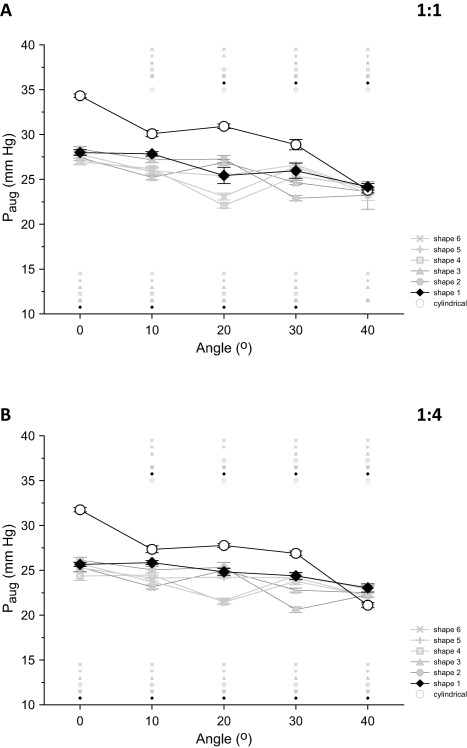
Average data (A) at frequency 1:1 and (B) at frequency 1:4, of the changes in diastolic “aortic” pressure augmentation (*P*_aug_) that took place when angle increased from 0 to 40° in increments of 10°. Data are shown separately for each IAB, each point is the average of 20 beats, and the error bars indicate standard deviations. For each IAB, the small matching symbols on top of each plot indicate statistically significant differences between 0° and each of the other angles. Similarly, the small matching symbols under each plot denote statistically significant differences compared to the cylindrical IAB. Statistical significance was assumed at *P* < 0.05.

The variations displayed by *P*_aug_ with changes in angle for all IABs are shown in Fig. 6A,B for frequencies 1:1 and 1:4, respectively.

Similarly to ΔedP, the magnitude of *P*_aug_ generated by the cylindrical IAB was progressively reduced with increasing angle for both assistance frequencies. Between 0° and 40, a 31% drop was observed at frequency 1:1 (23.8 ± 0.3 mm Hg at 40° vs. 34.3 ± 0.2 mm Hg at 0°, *P* < 0.05) and a 33% drop at frequency 1:4 (21.1 ± 0.3 mm Hg at 40° vs. 31.7 ± 0.2 mm Hg at 0°, *P* < 0.05).

Among all newly shaped IABs, the shape generally achieving the highest *P*_aug_ over all angles was *Shape 1*. This augmentation was not greater than the augmentation generated by the cylindrical IAB at any angle, except for 40°, but it displayed a more stable behavior with increasing angle, with a 14% decrease in *P*_aug_ at frequency 1:1 (24.2 ± 0.4 mm Hg at 40° vs. 28.0 ± 0.3 mm Hg at 0°, *P* < 0.05) and 11% decrease at frequency 1:4 (23.0 ± 0.5 mm Hg at 40° vs. 25.7 ± 0.2 mm Hg at 0°, *P* < 0.05) when angle was increased from 0 to 40°. Both for 1:1 and 1:4 *P*_aug_ with *Shape 1* was statistically significantly different from *P*_aug_ with the cylindrical IAB at all angles but one (40° at 1:1).

**Figure 7:**
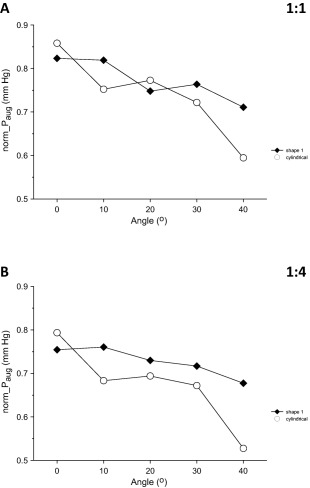
Changes in normalized diastolic “aortic” pressure augmentation (norm_*P*_aug_) when angle increased from 0 to 40° in increments of 10° (A) at frequency 1:1 and (B) at frequency 1:4. Data are shown for the cylindrical IAB and for *Shape 1*. Each point was derived by dividing the corresponding average *P*_aug_ with the nominal IAB volume.

After compensating for differences in nominal IAB volume, the superiority of *Shape 1* over the cylindrical IAB became more evident, especially at frequency 1:4 (Fig. 7A,B).

### Volumes displaced during inflation and deflation

For all IABs, the normalized values for the variations observed in *V*_infl_ and *V*_defl_ with changes in angle are listed in Table 1. Because of the large standard deviations, the statistical analysis performed on norm_*V*_infl_ and norm_*V*_defl_ could not yield any statistically significant differences between angles or IABs. As a result, these parameters were not pivotal in the determination of the best performing IABs during deflation and inflation, but rather were used to corroborate the decision that was made based on ΔedP and *P*_aug_. Indeed, *Shape 2* tended to generate larger norm_*V*_defl_ than the cylindrical IAB in both assistance frequencies and all angles, with the exception of 0° at 1:1 and 40° at 1:4. Similarly, *Shape 1* tended to produce the same or larger norm_*V*_infl_ compared to the cylindrical IAB in both frequencies and all angles, with the exception of 10° at 1:4.

**Table 1:** Normalized displaced volumes with support frequency 1:1 and 1:4

	Shape		0°	10°	20°	30°	40°
1:1	*Cylindrical*	norm_*V*_infl_	6 ± 5	8 ± 5	5 ± 4	8 ± 5	8 ± 6
		norm_*V*_defl_	4 ± 2	3 ± 2	2 ± 2	2 ± 2	2 ± 1
	*Shape 1*	norm_*V*_infl_	12 ± 10	11 ± 6	10 ± 5	10 ± 5	8 ± 5
		norm_*V*_defl_	4 ± 3	2 ± 1	3 ± 2	2 ± 2	3 ± 3
	*Shape 2*	norm_*V*_infl_	9 ± 7	6 ± 5	8 ± 4	16 ± 10	8 ± 5
		norm_*V*_defl_	4 ± 4	5 ± 3	3 ± 2	2 ± 2	3 ± 2
	*Shape 3*	norm_*V*_infl_	13 ± 8	7 ± 5	10 ± 9	13 ± 6	7 ± 8
		norm_*V*_defl_	4 ± 2	3 ± 2	3 ± 1	2 ± 2	2 ± 2
	*Shape 4*	norm_*V*_infl_	11 ± 10	8 ± 5	8 ± 6	10 ± 8	8 ± 6
		norm_*V*_defl_	5 ± 4	5 ± 3	3 ± 3	2 ± 2	3 ± 3
	*Shape 5*	norm_*V*_infl_	11 ± 8	15 ± 8	10 ± 6	6 ± 5	10 ± 8
		norm_*V*_defl_	4 ± 2	2 ± 3	2 ± 2	2 ± 2	4 ± 3
	*Shape 6*	norm_*V*_infl_	12 ± 7	8 ± 5	6 ± 5	9 ± 5	7 ± 4
		norm_*V*_defl_	3 ± 2	4 ± 2	4 ± 3	3 ± 2	2 ± 2
1:4	*Cylindrical*	norm_*V*_infl_	6 ± 5	7 ± 5	8 ± 6	8 ± 6	6 ± 4
		norm_*V*_defl_	3 ± 2	3 ± 2	3 ± 2	2 ± 2	3 ± 1
	*Shape 1*	norm_*V*_infl_	11 ± 9	7 ± 5	8 ± 5	9 ± 7	7 ± 6
		norm_*V*_defl_	5 ± 3	3 ± 2	2 ± 2	3 ± 3	2 ± 1
	*Shape 2*	norm_*V*_infl_	10 ± 6	7 ± 4	6 ± 5	15 ± 8	7 ± 4
		norm_*V*_defl_	4 ± 3	4 ± 2	4 ± 3	4 ± 3	3 ± 2
	*Shape 3*	norm_*V*_infl_	13 ± 8	6 ± 5	8 ± 5	9 ± 6	7 ± 6
		norm_*V*_defl_	5 ± 3	3 ± 2	3 ± 3	3 ± 3	3 ± 2
	*Shape 4*	norm_*V*_infl_	8 ± 6	8 ± 8	8 ± 5	11 ± 8	7 ± 5
		norm_*V*_defl_	4 ± 3	6 ± 3	3 ± 2	2 ± 2	2 ± 2
	*Shape 5*	norm_*V*_infl_	11 ± 7	6 ± 5	5 ± 4	8 ± 6	9 ± 5
		norm_*V*_defl_	4 ± 2	4 ± 2	3 ± 2	2 ± 2	4 ± 4
	*Shape 6*	norm_*V*_infl_	6 ± 4	9 ± 6	5 ± 3	8 ± 6	8 ± 6
		norm_*V*_defl_	5 ± 2	3 ± 2	5 ± 4	3 ± 2	3 ± 3

The normalized volumes are expressed as a percentage of the nominal IAB volume. norm_*V*_infl_: normalized volume pushed upstream the IAB tip during inflation; norm_*V*_defl_: normalized volume pulled from upstream the IAB tip during deflation.

## Discussion

### Summary of main findings

The hemodynamic performance of six novel shaped IABs was assessed and compared against that of a traditionally shaped cylindrical IAB in a mock circulation system at a range of operating angles. The absolute magnitude of parameters *P*_aug_ and ΔedP was gradually diminished with increasing angle for the cylindrical IAB, in agreement with published studies 6, 8. The performance of the cylindrical IAB was exceeded during deflation by *Shape 2*, even before adjusting for the different nominal volume between the two IABs. This shape consistently achieved, in terms of absolute magnitude, larger ΔedP at angles than the cylindrical IAB. Although the reduction in ΔedP of *Shape 2* was also gradually diminished with angle, it did so to a lesser degree than the cylindrical IAB; for example, this diminishment was only 53% (with frequency 1:1) and 40% (with frequency 1:4) of that of the cylindrical IAB, when angle increased from 0 to 40°. During inflation *Shape 1* displayed a more stable behavior with increasing angle compared to the cylindrical IAB; for example, with an increase in angle from 0 to 40°, diastolic “aortic” pressure augmentation dropped only by 45% (with frequency 1:1) and by 33% (with frequency 1:4) of the drop reached with the cylindrical IAB. After normalizing with respect to the nominal volume of each IAB, *Shape 1* generally achieved higher *P*_aug_ over most angles.

### Rationale for the new design

At the horizontal position a cylindrical IAB inflates and deflates uniformly along its length. However, at angled positions inflation starts from the tip and progresses toward the base, while deflation progresses in reverse order 5, 6, 7. This is due to the hydrostatic pressure difference along the IAB length; inflation starts from the IAB end that is surrounded by lower liquid pressure, and deflation starts from the IAB end that is surrounded by higher liquid pressure 23.

During inflation of a cylindrical IAB at an angle, fluid is displaced progressively along the length of the IAB, as the IAB membrane inflates from tip to base. This displaced fluid will encounter more opposition traveling toward the tip than toward the base of the IAB (as the former is already inflated, obstructing the passage upstream, while the latter is still deflated) and as a result fluid will be pushed gradually toward the base of the IAB. Therefore, the amount of fluid pushed upstream the tip in the retrograde direction is reduced compared to the horizontal position 6. Following the same reasoning, during deflation of a cylindrical IAB at an angle, fluid is sucked progressively along the length of the IAB, as the IAB membrane deflates from base to tip. This suction will encounter more opposition pulling fluid from the tip than from the base of the IAB (as the former is still inflated, blocking the passage upstream, whilst the latter is already deflated). As a result, fluid will be gradually pulled from the base of the IAB and the amount of fluid that is pulled from upstream the tip in the antegrade direction, is reduced compared to the horizontal position 6. This reduction in the amount of fluid that is displaced upstream the IAB tip, in the retrograde direction during inflation and in the antegrade direction during deflation, can explain the deterioration in IABP hemodynamic performance observed at angles.

When IABs with oblance or lance configuration operate at an angle, inflation will also start from the tip and progress toward the base, while deflation will start from the base and progress toward the tip; because this pattern is induced by the hydrostatic pressure difference along the IAB membrane, it is reasonable to assume that the same mechanism will be active irrespective of the shape of the IAB membrane.

During inflation of a lance IAB at an angle, similarly to a cylindrical IAB, fluid will be displaced progressively along the length of the IAB, as the IAB membrane gradually inflates from tip to base. Contrary to the cylindrical IAB however, while inflation is propagating along the conical part, this displaced fluid will, in effect, be driven evenly toward the tip and the base of the IAB, until inflation reaches the cylindrical segment, where a switch to the displacement pattern observed with the cylindrical IAB can be expected. Thus overall, a lance configuration inflated at an angle should be able to push more fluid in the retrograde direction upstream the tip of the IAB during early inflation (and the same amount of fluid during late inflation) compared to a cylindrical IAB. During deflation of a lance IAB at an angle, similarly to a cylindrical IAB, fluid will be sucked progressively along the length of the IAB, as the IAB membrane deflates from base to tip. Also similarly to the cylindrical IAB, while deflation propagates along the cylindrical segment, this suction will encounter more opposition pulling fluid from the tip than from the base of the IAB and as a result fluid will be gradually pulled from the base of the IAB during the early part of deflation. This pattern however changes during the latter part of deflation, when the conical segment of the IAB starts to deflate; the suction will, in effect, pull fluid evenly from the tip and the base of the IAB. Thus overall, a lance configuration deflated at an angle should be able to pull more fluid in the antegrade direction upstream the tip of the IAB during late deflation (and the same amount of fluid during early deflation) compared to a cylindrical IAB. Our results confirm this expectation, indicating the lance configuration with the longest conical segment (*Shape 2*) as the new IAB design that performed optimally during the deflation phase, according to the ΔedP results.

The design rationale behind the oblance configurations is rather different. With the cylindrical segment located at the tip of the IAB, the advantage the lance configuration had over the cylindrical IAB during early inflation and late deflation might be lost. The potential advantage of the oblance design lies on the size of the cylindrical tip, which has larger diameter than the tip of the cylindrical IAB. It is possible that when the very tip of the oblance design inflates at the onset of inflation and deflates at the end of deflation, due to size alone, it will push more fluid in the retrograde direction and pull more fluid in the antegrade direction upstream the tip, compared to the cylindrical IAB. This prediction seems to be confirmed by our results for the inflation phase, because the oblance configuration with the longest conical segment (*Shape 1*) was found to provide the most promising outcome among the newly shaped IABs, according to the *P*_aug_ results.

### Clinical implications

IABs with shapes that deviate from the traditional cylindrical shape were found to have improved hemodynamic performance at angles compared to a cylindrical IAB, when tested in vitro. If these results are confirmed by in vivo testing, they would provide evidence that IAB counterpulsation therapy may be delivered more efficiently to patients nursed at the semirecumbent position when these newly shaped IABs are used. The fact that the new shape that performed better at inflation (based on parameter *P*_aug_) was different from the new shape that performed better at deflation (based on parameter ΔedP) suggests that different patient groups might benefit from different IAB shapes. For example, a patient receiving IABP support as a prophylactic measure during a high‐risk coronary procedure might benefit more by improved inflation performance, which would ensure enhanced coronary blood flow augmentation (*Shape 1*). Oppositely, a patient in cardiogenic shock might benefit more by improved deflation performance, which would in turn provide boosted LV unloading (*Shape 2*). These findings promote the idea of personalized rather than generalized patient therapy for the achievement of higher IABP therapeutic efficiency, with a choice of IAB shape that prioritizes the recovery of those hemodynamic indices that are more in need of support in the unassisted circulation.

### Limitations

A cylindrical IAB of the same size as the newly shaped IABs was not available, and normalization, with respect to the nominal IAB volume, was carried out in order to compensate for the fact that the newly shaped IABs were smaller than the cylindrical IAB. The nominal volume was assumed to be the volume the IABs were able to displace when inflated at transmural pressure of 110 mm Hg, which is considered a realistic clinical assumption.

The mean “aortic” pressure in the MCS was pathophysiological, falling within the hypertensive spectrum. Nonetheless, it is not anticipated that the observed trends for any of the hemodynamic parameters of interest would be different for a more physiological mean *P*; when tested statically, the volumes displaced by each of the IAB membranes during inflation were linear (and parallel between them) functions of transmural pressure over a wide range of transmural pressures.

A final limitation of the present experimental work is the lack of “coronary” flow measurements, as an additional indicator of inflation performance. Because of technical constraints, the MCS did not mimic the mechanism of coronary systolic flow impediment; hence coronary flow quantification would not be meaningful.

## Conclusion

Intra‐aortic balloon pumps with new shapes were less affected, compared to a cylindrical IAB, when operated at a clinical range of angles to the horizontal. The hemodynamic performance of the cylindrical IAB was exceeded during deflation by *Shape 2*, which was found to consistently achieve, in terms of absolute magnitude, larger reduction in end‐diastolic pressure at angles than the cylindrical IAB. Although this reduction was gradually diminished with angle, it did so to a lesser degree than the cylindrical IAB; this diminishment was only 53% (with frequency 1:1) and 40% (with frequency 1:4) of that of the cylindrical IAB, when angle increased from 0 to 40°. During inflation *Shape 1* displayed a more stable behavior with increasing angle compared to the cylindrical IAB; with an increase in angle from 0 to 40°, diastolic “aortic” pressure augmentation dropped only by 45% (with frequency 1:1) and by 33% (with frequency 1:4) of the drop reached with the cylindrical IAB. After compensating for differences in nominal IAB volume, this shape generally achieved higher augmentation in diastolic “aortic” pressure over most angles.

These newly shaped IABs could allow for IABP therapy to become more efficient for patients nursed at the semirecumbent position. The fact that the new shape that performed better at inflation was different from the new shape that performed better at deflation suggests that different patient groups might benefit from different IAB shapes.

These findings promote the idea of personalized rather than generalized patient therapy for the achievement of higher IABP therapeutic efficiency, with a choice of IAB shape that prioritizes the recovery of those hemodynamic indices which are more in need of support in the unassisted circulation.

**Conflict of Interest:** None.
